# A Six-Gene Signature Predicts Survival of Patients with Localized Pancreatic Ductal Adenocarcinoma

**DOI:** 10.1371/journal.pmed.1000307

**Published:** 2010-07-13

**Authors:** Jeran K. Stratford, David J. Bentrem, Judy M. Anderson, Cheng Fan, Keith A. Volmar, J. S. Marron, Elizabeth D. Routh, Laura S. Caskey, Jonathan C. Samuel, Channing J. Der, Leigh B. Thorne, Benjamin F. Calvo, Hong Jin Kim, Mark S. Talamonti, Christine A. Iacobuzio-Donahue, Michael A. Hollingsworth, Charles M. Perou, Jen Jen Yeh

**Affiliations:** 1 Department of Pharmacology, University of North Carolina at Chapel Hill, Chapel Hill, North Carolina, United States of America; 2 Department of Surgery and Robert H. Lurie Comprehensive Cancer Center, Northwestern University, Feinberg School of Medicine, Chicago, Illinois, United States of America; 3 The Eppley Cancer Institute, University of Nebraska, Omaha, Nebraska, United States of America; 4 Lineberger Comprehensive Cancer Center, University of North Carolina at Chapel Hill, Chapel Hill, North Carolina, United States of America; 5 Department of Pathology, University of North Carolina at Chapel Hill, Chapel Hill, North Carolina, United States of America; 6 Department of Statistics and Operations Research, University of North Carolina at Chapel Hill, Chapel Hill, North Carolina, United States of America; 7 Department of Surgery, University of North Carolina at Chapel Hill, Chapel Hill, North Carolina, United States of America; 8 Department of Surgery, NorthShore University HealthSystem, Baltimore, Maryland, United States of America; 9 Department of Pathology, The Johns Hopkins Medical Institutions, Baltimore, Maryland, United States of America; 10 Department of Genetics, University of North Carolina at Chapel Hill, Chapel Hill, North Carolina, United States of America; Fred Hutchinson Cancer Research Center, United States of America

## Abstract

**Background:**

Pancreatic ductal adenocarcinoma (PDAC) remains a lethal disease. For patients with localized PDAC, surgery is the best option, but with a median survival of less than 2 years and a difficult and prolonged postoperative course for most, there is an urgent need to better identify patients who have the most aggressive disease.

**Methods and Findings:**

We analyzed the gene expression profiles of primary tumors from patients with localized compared to metastatic disease and identified a six-gene signature associated with metastatic disease. We evaluated the prognostic potential of this signature in a training set of 34 patients with localized and resected PDAC and selected a cut-point associated with outcome using X-tile. We then applied this cut-point to an independent test set of 67 patients with localized and resected PDAC and found that our signature was independently predictive of survival and superior to established clinical prognostic factors such as grade, tumor size, and nodal status, with a hazard ratio of 4.1 (95% confidence interval [CI] 1.7–10.0). Patients defined to be high-risk patients by the six-gene signature had a 1-year survival rate of 55% compared to 91% in the low-risk group.

**Conclusions:**

Our six-gene signature may be used to better stage PDAC patients and assist in the difficult treatment decisions of surgery and to select patients whose tumor biology may benefit most from neoadjuvant therapy. The use of this six-gene signature should be investigated in prospective patient cohorts, and if confirmed, in future PDAC clinical trials, its potential as a biomarker should be investigated. Genes in this signature, or the pathways that they fall into, may represent new therapeutic targets.

*Please see later in the article for the Editors' Summary*

## Abstract 2

Jen Jen Yeh and colleagues developed and validated a six-gene signature in patients with pancreatic ductal adenocarcinoma that may be used to better stage the disease in these patients and assist in treatment decisions.

## Editors' Summary

**Background:**

Pancreatic cancer kills nearly a quarter of a million people every year. It begins when a cell in the pancreas (an organ lying behind the stomach that produces digestive enzymes and hormones such as insulin, which controls blood sugar levels) acquires genetic changes that allow it to grow uncontrollably and to spread around the body (metastasize). Nearly all pancreatic cancers are “pancreatic ductal adenocarcinomas” (PDACs)—tumors that start in the cells that line the tubes in the pancreas that take digestive juices to the gut. Because PDAC rarely causes any symptoms early in its development, it has already metastasized in about half of patients before it is diagnosed. Consequently, the average survival time after a diagnosis of PDAC is only 5–8 months. At present, the only chance for cure is surgical removal (resection) of the tumor, part of the pancreas, and other nearby digestive organs. The operation that is needed for the majority of patients—the Whipple procedure—is only possible in the fifth of patients whose tumor is found when it is small enough to be resectable but even with postoperative chemotherapy, these patients only live for 23 months after surgery on average, possibly because they have micrometastases at the time of their operation.

**Why Was This Study Done?:**

Despite this poor overall outcome, about a quarter of patients with resectable PDAC survive for more than 5 years after surgery. Might some patients, therefore, have a less aggressive form of PDAC determined by the biology of the primary (original) tumor? If this is the case, it would be useful to be able to stratify patients according to the aggressiveness of their disease so that patients with very aggressive disease could be given chemotherapy before surgery (neoadjuvant therapy) to kill any micrometastases. At present neoadjuvant therapy is given to patients with locally advanced, unresectable tumors. In this study, the researchers compare gene expression patterns in primary tumor samples collected from patients with localized PDAC and from patients with metastatic PDAC between 1999 and 2007 to try to identify molecular markers that distinguish between more and less aggressive PDACs.

**What Did the Researchers Do and Find?:**

The researchers identified a six-gene signature that was associated with metastatic disease using a molecular biology approach called microarray hybridization and a statistical method called significance analysis of microarrays to analyze gene expression patterns in primary tumor samples from 15 patients with localized PDAC and 15 patients with metastatic disease. Next, they used a training set of tumor samples from another 34 patients with localized and resected PDAC, microarray hybridization, and a graphical method called X-tile to select a combination of expression levels of the six genes that discriminated optimally between high-risk (aggressive) and low-risk (less aggressive) tumors on the basis of patient survival (a “cut-point”). When the researchers applied this cut-point to an independent set of 67 tumor samples from patients with localized and resected PDAC, they found that 42 patients had high-risk tumors. These patients had an average survival time of 15 months; 55% of them were alive a year after surgery. The remaining 25 patients, who had low-risk tumors, had an average survival time of 49 months and 91% of them were alive a year after resection.

**What Do These Findings Mean?:**

These and other findings identify a six-gene signature that can predict outcomes in patients with localized, resectable PDAC better than, and independently of, established clinical markers of outcome. If the predictive ability of this signature can be confirmed in additional patients, it could be used to help patients make decisions about their treatment. For example, a patient wondering whether to risk the Whipple procedure (2%–6% of patients die during this operation and more than 50% have serious postoperative complications), the knowledge that their tumor was low risk might help them decide to have the operation. Conversely, a patient in poor health with a high-risk tumor might decide to spare themselves the trauma of major surgery. The six-gene signature might also help clinicians decide which patients would benefit most from neoadjuvant therapy. Finally, the genes in this signature, or the biological pathways in which they participate, might represent new therapeutic targets for the treatment of PDAC.

**Additional Information:**

Please access these Web sites via the online version of this summary at http://dx.doi.org/10.1371/journal.pmed.1000307.

- The US National Cancer Institute provides information for patients and health professionals about all aspects of pancreatic cancer (in English and Spanish), including a booklet for patients
- The American Cancer Society also provides detailed information about pancreatic cancer
- The UK National Health Service and Cancer Research UK include information for patients on pancreatic cancer on their Web sites
- MedlinePlus provides links to further resources on pancreatic cancer (in English and Spanish)
- Cure Pancreatic Cancer provides information about scientific and medical research related to the diagnosis, treatment, cure, and prevention of pancreatic cancer
- Pancreatic Cancer Action Network is a US organization that supports research, patient support, community outreach, and advocacy for a cure for pancreatic cancer

## Introduction

Pancreatic ductal adenocarcinoma (PDAC), comprising over 90% of all pancreatic cancers, remains a lethal disease with an estimated 232,000 new cases, 227,000 deaths per year worldwide, and a less than 5% 5-y survival rate [1],[2]. Currently the standard of care for the 20% of patients with localized disease is surgery followed by chemotherapy, and in some cases radiation. Unfortunately, despite the use of adjuvant therapy, median survival remains at best 23 mo [3]. It is important to note, however, that up to 27% of patients with resected PDAC can survive for 5 y [4]–[10]. However, in studies examining actual long-term survivors [4]–[10], only two have found that adjuvant therapy was associated with improved survival [9],[10]. In addition, randomized controlled trials of gemcitabine-based chemotherapy demonstrate an improvement in median survival of at best 3 mo [3],[11]. One possible conclusion from these studies is that tumor biology dictates outcome and that our current adjuvant therapy has only a modest impact on altering a patient's course.

Hypothesizing that the dismal outcome of patients with localized disease is due to the presence of micrometastatic disease, current clinical investigation has focused on preoperative or neoadjuvant therapy [12],[13]. This approach, in which patients who cannot tolerate the stress of therapy or who develop metastatic disease during treatment are spared surgery, has demonstrated an overall survival of 34 mo in this highly selected patient population [12],[13]. Therefore the ability to select patients who would most benefit from a neoadjuvant approach may be important. One way to select these individuals is to define a prognostic gene signature that can identify patients with more aggressive tumor biology upfront.

Expression profiling of PDAC has lead to further studies of additional molecular diagnostic and prognostic markers [14]–[19]. However, the search for genes of biological significance in these large datasets continues to be challenging. One approach to identify genes or pathways that are biologically relevant is to study those that are of prognostic significance [20]. Lowe and colleagues found differential gene expression changes associated with nodal status in primary PDAC [21], suggesting that molecular differences in primary PDAC do exist. We hypothesized that by comparing primary PDAC tumors at the extremes of disease, we would identify molecular changes reflective of differences in biology within primary PDAC tumors.

## Methods

### Patients

PDAC samples from 15 patients with resected primary PDAC from the University of North Carolina at Chapel Hill (UNC) and 15 patients with metastatic PDAC from the University of Nebraska Medical Center Rapid Autopsy Pancreatic Program (NEB) were used to derive differentially expressed genes associated with metastatic disease. For the NEB samples, human pancreatic tumors from decedents who had previously been diagnosed with PDAC, and who generously consented to post mortem examinations, were obtained from the institutional review board (IRB)-approved NEB Tissue Bank. To ensure minimal degradation of tissue, organs were harvested within 3 h post mortem and the specimens flash frozen in liquid nitrogen.

The training cohort included 34 patients with resected PDAC from Johns Hopkins Medical Institutions (JHMI). The testing or validation cohort included patients from two institutions: 48 from Northwestern Memorial Hospital (NW) and 19 from NorthShore University HealthSystem (NSU). All samples were collected between 1999 and 2007 at the time of operation and flash frozen in liquid nitrogen after approval by each individual IRB. The UNC IRB approved use of all de-identified samples for this study. All available samples were reviewed by a single pathologist (KAV). De-identified data including tumor, node, and metastasis (TNM), grade or differentiation, margin status, and survival were available for the majority of patients.

### RNA Isolation and Microarray Hybridization

All RNA isolation and hybridization was performed on Agilent (Agilent Technologies) human whole genome 4×44 K DNA microarrays and at UNC. RNA was extracted from macrodissected snap-frozen tumor samples using Allprep Kits (Qiagen) and quantified using nanodrop spectrophotometry (ThermoScientific). RNA quality was assessed with the use of the Bioanalyzer 2100 (Agilent Technologies). RNA was selected for hybridization using RNA integrity number and by inspection of the 18S and 28S ribosomal RNA. Similar RNA quality was selected across samples. One microgram of RNA was used as a template for DNA preparations and hybridized to Agilent 4×44 K whole human genome arrays (Agilent Technologies). cDNA was labeled with Cy5-dUTP and a reference control (Stratagene) was labeled with Cy3-dUTP using the Agilent (Agilent Technologies) low RNA input linear amplification kit and hybridized overnight at 65°C to Agilent 4×44 K whole human genome arrays (Agilent Technologies). Arrays were washed and scanned using an Agilent scanner (Agilent Technologies). The data are publicly available in Gene Expression Omnibus database (http://www.ncbi.nlm.nih.gov/geo/query/acc.cgi?acc=GSE21501).

### Microarray and Statistical Analysis

All array data were normalized using Lowess normalization. Data were excluded for genes with poor spot quality or genes that did not have mean intensity greater than 10 for one of the two channels (green and red) in at least 70% of the experiments. The log_2_ ratio of the mean red intensity over mean green intensity was calculated for each gene and went through LOWESS normalization [22]. Missing data were imputed using the k-nearest neighbors imputation (KNN) with k = 10 [23]. A distance weighted discrimination (DWD) was used to detect the systematic biases between the different datasets and then global adjustments made to remove these biases [24]. Genes that were significantly up- or down-regulated were identified using significance analysis of microarrays (SAM) [25]. Two centroids were created using the mean gene expression profile of this significant gene list from the derivation set and used to develop a single sample predictor (SSP, nearest centroid algorithm) [26] for an objective classifier. After DWD, the SSP was applied to a 34-patient training set where any new sample was compared to the resected centroid and assigned by the SSP distance function to the resected centroid using (1 − Pearson correlation coefficient). The X-Tile software program, which assigns a two-population log-rank value to each sample and then determines the best cut-point, was used to determine the best threshold for classifying samples into high- and low-risk categories [27]. X-Tile predicted that the (1− Pearson correlation coefficient) distance of 1 would be the appropriate cut-point to stratify patients into a high- and low-risk group (*p* = 0.006). A second independent validation cohort was then used as a test set using this predetermined cut-point to evaluate outcome.

Survival analysis was performed using the statistical software programs R, the R-package “survival,” and SPSS (SPSS, Inc.). Overall survival (OS) was analyzed using the Kaplan-Meier product-limit method and the significance of our variables was measured by the log-rank test. The Fisher exact test was used to analyze associations between two variables, the Pearson Chi-square test was used to analyze association between more than two variables. Multivariable analysis and analysis of continuous and ordinal variables was performed using the Cox proportional hazards regression method.

### Tissue Microarrays

Tissue microarrays (TMAs; UNC2) were prepared from formalin-fixed paraffin-embedded tissue sections using a 2-mm punch. The arrays contained triplicate cores of matched normal and tumor tissue as well as chronic pancreatitis when available, from each patient. We prepared 5-µm sections from each TMA block. Hematoxylin and eosin stained slides from each TMA block were reviewed by a pathologist (KAV) to ensure that tissues were cored accurately.

### Immunohistochemistry

Slides with 5-µM sections from the paraffin-embedded specimens were deparaffinized and rehydrated. The slides were then subjected to alkaline heat antigen-retrieval using 1% Tris EDTA for 20 min in a steamer. All slides were incubated with 3% H_2_O_2_ for 5 min and washed with TBS. The slides were further treated with protein block solution (bovine serum albumin) for 20 min. The sections were incubated with primary KLF6 1∶150 antibody (sc-7158, Santa Cruz Biotechnology) for 60 min at room temperature. Following a TBS wash, the slides were incubated with secondary labeled Polymer-HRP anti-rabbit (Dako K4002) for 30 min. This was followed by a 5-min incubation with the substrate-chromogen, 3,3′-diaminobenzidine (Vector SK-4100). The sections were counterstained with Harris hematoxylin. Positive KLF6 staining was defined as when more than 5% of cells expressed the marker and graded from 0 (no staining) to 4 (strong staining). The results of each protein marker were then expressed as intensity (I) and proportion (P) of positive epithelial cells and the score as the product of I and P [28],[29]. All stained slides were reviewed in a blinded fashion (JMA).

## Results

### Patient and Tumor Characteristics

In order to study the extremes of PDAC tumor biology, we collected a diverse set of resected PDAC specimens from patients with and without metastases. As the tumor microenvironment is increasingly recognized to play a critical role in tumorigenesis [30]–[33], tissues were macrodissected in order to preserve the normal adjacent tissue and stroma of the tumors. The characteristics of the dataset used to derive the signature (derivation set) comprised 15 primary resected PDAC tumors (UNC1) and 15 primary tumors from patients with metastatic PDAC (NEB). The training set comprised 34 patients with primary PDAC and the independent validation test set comprised 67 patients with primary PDAC (Tables 1 and 2). There were no differences in RNA quality between the decedent and resected PDAC samples. Available treatment data of the patients in the training and test sets are also shown. One of 15 (7%) UNC1 patients received preoperative or neoadjuvant chemotherapy and 11/15 (73%) NEB patients received chemotherapy less than 6 mo prior to death. No patient in the 34-patient training set received neoadjuvant chemotherapy. Only 3% (2/67) of patients in the test set received neoadjuvant chemotherapy and 45% (30/67) of patients received postoperative or adjuvant chemotherapy.

**Table 1:** Patient, tumor, and treatment characteristics in the derivation set.

Demographics (Derivation Set)	NEB, *n* = 15	UNC1, *n* = 15
**Median follow-up (mo)**	NA	6 (1–35)
**T stage**		
1	NA	—
2	NA	2 (13%)
3	NA	12 (80%)
4	NA	1 (7%)
**N stage**		
0	NA	7 (47%)
1	NA	8 (53%)
**M stage**		
0	0	15 (100%)
1	15	0
**Grade**		
1	NA	2 (14%)
2	NA	8 (57%)
3	NA	4 (29%)
**Margin**		
Negative	NA	12 (80%)
Positive	NA	3 (20%)
**Neoadjuvant therapy**		
No	NA	14 (93%)
Yes	NA	1 (7%)
**Adjuvant therapy**		
No	NA	11 (73%)
Yes	NA	4 (27%)
**Chemotherapy**		
No	3 (20%)	NA
Yes	12 (80%)	NA
**Median survival (mo)**	NA	9 (1–35)

NA, not available.

**Table 2:** Patient, tumor, and treatment characteristics in the training and testing sets.

Demographics	JHMI (Training Set), *n* = 34	NW/NSU (Testing Set),*n* = 67	UNC2 (TMA), *n* = 50
**Median follow-up (mo)**	14 (2–54)	17 (2–59)	11 (0–51)
**T stage**			
1	—	2 (3%)	5 (10%)
2	6 (18%)	10 (16%)	8 (16%)
3	27 (79%)	51 (81%)	32 (66%)
4	1 (3%)	—	4 (8%)
**N stage**			
0	2 (6%)	25 (38%)	15 (31%)
1	32 (94%)	41 (62%)	34 (69%)
**M stage**			
0	34 (100%)	67 (100%)	47 (96%)
1	0	0	2 (6%)
**Grade**			
1	1 (3%)	2 (3%)	2 (4%)
2	13 (38%)	34 (54%)	26 (54%)
3	20 (59%)	27 (43%)	20 (42%)
**Margin**			
Negative	NA	51 (80%)	7 (78%)
Positive	NA	13 (20%)	2 (12%)
**Neoadjuvant therapy**			
No	34 (100%)	65 (97%)	7 (88%)
Yes	0	2 (3%)	1 (12%)
**Adjuvant therapy**			
No	NA	30 (45%)	NA
Yes	NA	37 (55%)	NA
**Median survival (mo)**	13 (2–54)	21 (3–59)	12 (0–51)

NA, not available.

### Gene Expression Differences in Nonmetastatic And Metastatic Primary Tumors

**Figure 1:**
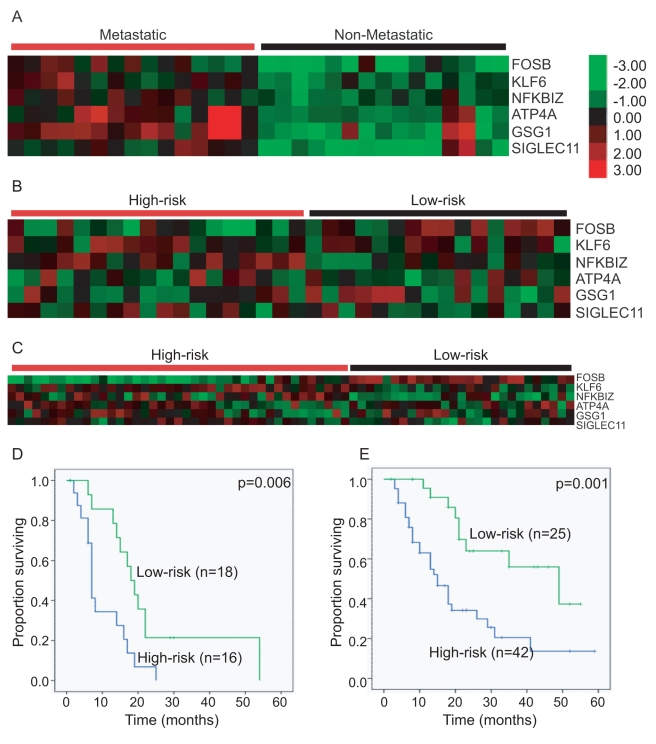
Identification, development, and application of a six-gene signature for PDAC. Clustering of (A) the six genes defined by SAM evaluation of the metastatic compared to nonmetastatic primary PDAC using a false discovery rate of 5%; (B) patient samples into high- and low-risk groups in a training set of 34 patients with localized and resected PDAC using the X-tile determined cut-point of a Pearson correlation coefficient of zero; (C) patient samples into high- and low-risk groups in an independent test set of 67 patients with localized and resected PDAC using the predetermined cut-point of zero. Kaplan-Meier overall survival of (D) the training set classified into high- and low-risk groups according to the X-tile determined cut-point of a Pearson correlation coefficient of zero; (E) and the independent test set classified into high- and low-risk groups according to the same predetermined cut-point.

We hypothesized that we could enrich for molecular differences in primary PDAC, which may be clinically and biologically relevant, through examining primary tumors representing opposite spectrums of PDAC: early (localized) and late (metastatic) stage. To accomplish this, we compared nonmetastatic (UNC1) with metastatic (NEB) primary PDAC tumors. As the methods of procurement for these tumors differed, we used DWD to identify systematic biases between the two datasets [24]. This method has been used previously to successfully combine three breast cancer datasets across three microarray platforms [26], across species [34], and across multiple datasets [35],[36]. We therefore used DWD to adjust for the systematic biases between the UNC1 and NEB datasets by taking advantage of the fact that each dataset also had 15 normal pancreas samples assayed. In short, we used DWD to adjust these 15 tumor-normal pairs from both datasets to have similar distributions in principal component (PC) 1×PC 2 space. After the DWD adjustment, we used SAM to identify differentially expressed genes [22],[25]. Using a false discovery rate of 5%, we identified six genes that were differentially overexpressed between nonmetastatic and metastatic primary tumors: FBJ murine osteosarcoma viral oncogene homolog B (*Fos B*), Kruppel-like factor 6 (*KLF6*), nuclear factor of kappa light polypeptide gene enhancer in B-cells inhibitor, zeta (*NFKBIZ*, *IKBZ*, *MAIL*), ATPase H+/K+ exchanging, alpha polypeptide (*ATP4A*), germ cell associated 1 (*GSG1*), and sialic acid binding Ig-like lectin 11 (*SIGLEC11*) (Figure 1A; Table S1).

### Development of a Classifier Using the Six-Gene Signature

We examined the relationship of our six-gene signature to outcome using a training set of 34 patients with localized and resected PDAC. After identifying and adjusting for systematic bias using DWD [24], a resected centroid-based predictor [26] was created using the 30 samples in the derivation dataset. The centroid was then applied to the DWD-adjusted training set of primary PDAC patients to determine the performance of the six-gene signature. X-tile [27] was used to determine the optimal distance function to the centroid cut-point for classifying this training set of patients into high-risk and low-risk groups on the basis of survival (Figure 1B and 1D). The optimal cut-point occurred at a Pearson correlation coefficient of zero (*p* = 0.006) with patients with Pearson correlation coefficients greater than zero in the low-risk and less than zero in the high-risk groups.

### Application of the Six-Gene Signature to an Independent Validation Cohort of 67 Patients

In order to evaluate the performance of the cut-point determined by X-tile [27], we applied the cut-point to an independent validation test set of 67 patients with primary PDAC. Our predetermined Pearson correlation coefficient cut-point of zero distance to the centroid successfully stratified patients into high- (*n* = 42) and low-risk groups (*n* = 25) with a median overall survival (OS) of 15 versus 49 mo (*p* = 0.001) (Figure 1C and 1E). Patients in the high-risk group had 1-, 2-, and 3-y estimated survival rates of 55%, 34%, and 21%, compared to 91%, 64%, and 56% in the low-risk group.

Previous studies in PDAC have found that nodal status is the most predictive of outcome for patients with localized PDAC [37]. We compared our prognostic signature to current clinical prognostic benchmarks. We found that tumors that were node positive (*p* = 0.091) and grade 2 or 3 trended towards a shorter survival (*p* = 0.080). Neither T stage (*p* = 0.977) nor margin status (*p* = 0.223) were prognostic in this cohort. Treatment with adjuvant chemotherapy (*p* = 0.699) or with neoadjuvant chemotherapy (*p* = 0.409) was also not prognostic, although only two patients received neoadjuvant chemotherapy. We found no gene expression changes between the tumors of the two patients who received neoadjuvant chemotherapy and the tumors of patients who received no treatment prior to surgery.

An important feature of any prognostic signature is that it should be independent or additive to currently used clinicopathologic prognostic criteria. We therefore compared the prognostic importance of our molecular signature in the setting of grade (*p* = 0.417), nodal status (*p* = 0.381), T stage (*p* = 0.675), and margin status (*p* = 0.295). We found that our six-gene signature was the only independent predictor of survival in the 57 patients with complete data, with a hazard ratio of 4.1 (95% confidence interval 1.7–10.0) (Table 3).

**Table 3:** Cox proportional hazards regression analysis of the six-gene signature.

Variable	Hazard Ratio	CI	*p*-Value
**Six-gene signature**	4.1	1.7–10.0	0.002
**T stage**	—	—	0.675
**N stage**	—	—	0.381
**Grade**	—	—	0.417
**Margin status**	—	—	0.295

CI, confidence interval.

We also looked at whether our six-gene signature was confounded by available clinicopathological variables. We found no association between our molecular signature, and tumor size, grade, margin status, nodal status, and neoadjuvant or adjuvant chemotherapy in our independent test set (Table 4).

**Table 4:** Relationship between the six-gene signature and clinicopathological variables.

Variable	Six-Gene Signature		
	High Risk	Low Risk	*p*-Value
**T stage**			
1	1 (50%)	1 (50%)	0.886
2	6 (60%)	4 (40%)	—
3	33 (65%)	18 (35%)	—
**N stage**			
0	13 (52%)	12 (48%)	0.203
1	28 (68%)	13 (32%)	—
**Grade**			
1	1 (50%)	1 (50%)	0.788
2	22 (65%)	12 (35%)	—
3	19 (70%)	8 (30%)	—
**Margin**			
Negative	31 (59%)	22 (41%)	0.344
Positive	9 (75%)	3 (25%)	—
**Neoadjuvant therapy**			
No	42 (65%)	23 (35%)	0.136
Yes	0 (0%)	2 (100%)	—
**Adjuvant therapy**			
No	24 (65%)	13 (35%)	0.801
Yes	18 (60%)	12 (40%)	—

### KLF6 Expression in Primary PDAC

**Figure 2:**
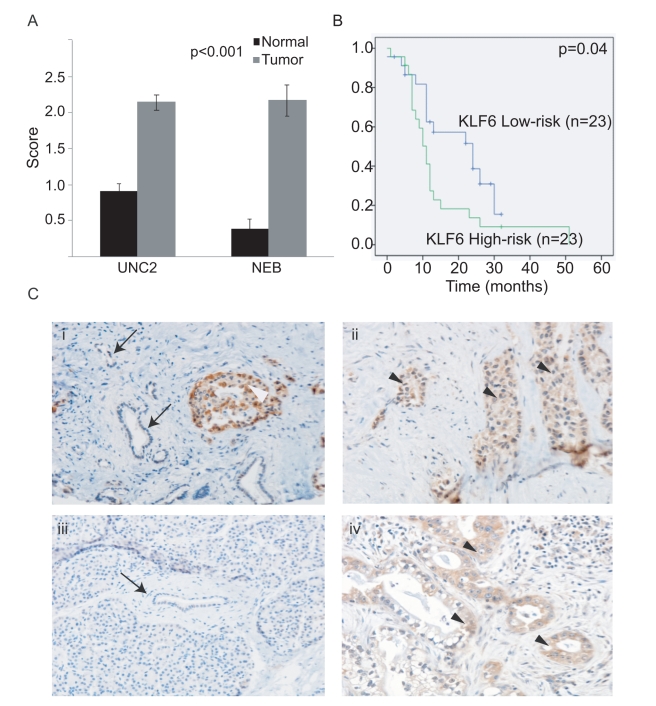
Significance of KLF6 and Fos B expression in primary PDAC. (A) KLF6 staining is significantly higher in PDAC compared to normal adjacent pancreas in an independent dataset of a 50-patient TMA (UNC2) as well as NEB samples used for the original analysis. (B) Kaplan-Meier overall survival of 50 patients classified by high and low KLF6 scores, using the median cutoff score of 1.5. (C) KLF6 immunostaining in the primary tumor of a patient who died of metastatic disease (ii) and in a resected primary tumor (iv). Minimal staining is seen in the matched normal adjacent tissue of both patients (i, iii). KLF6 immunostaining in islet cells (i, white arrowhead). Arrows illustrate normal ductal epithelium. Black arrowheads illustrate tumor.

In order to further validate the six-gene signature, we performed immunohistochemical analyses for KLF6, which showed a wide range of expression values between nonmetastatic versus metastatic samples (Figure 1A). To evaluate KLF6 protein expression, we obtained another independent dataset of 50 patients represented on a TMA with matched normal, chronic pancreatitis, and PDAC (UNC2, Table 2). First, using the median score of 1.5 as the cutoff, we found that KLF6 expression was much higher in tumors compared to normal pancreas (*p*<0.001) (Figure 2A and 2C). KLF6 expression was strong in normal islet cells in agreement with a previous study (Figure 2Ci, white arrowhead) [38]. Second, we found that KLF6 expression with a score greater than 1.5 (high) was associated with a shorter median survival of 11 mo compared to 24 mo for patients with KLF6 expression scores less than 1.5 (low) (*p* = 0.04) (Figure 2B).

## Discussion

We profiled and compared nonmetastatic and metastatic primary PDAC tumors and identified a six-gene signature. Although this signature was not derived on the basis of outcomes, we show that it was prognostic in a true test set of resectable PDAC patients. Importantly, our six-gene signature was independently predictive of survival, stratifying patients with median survivals of 15 compared to 49 mo, outperforming current pathological staging criteria, suggesting that our signature will be a powerful prognostic tool for patients with localized PDAC.

PDAC continues to be a devastating disease with few long-term survivors. Surgery remains the standard therapy for patients diagnosed with resectable PDAC [39]. Yet with a median survival only of less than 2 y after surgery, the attendant postoperative mortality rate of 2%–6% [40],[41], and postoperative complication and hospital readmission rates of 59% [41],[42], the decision for surgery should be made cautiously. Therefore, improved patient selection for therapy is necessary. For the majority of patients who cannot undergo surgery, gemcitabine chemotherapy remains the best option, yet only 5%–10% of patients respond to the treatment [43],[44]. Given the current therapeutic limitations, additional prognostic tools are needed to help a patient decide whether to have surgery, and/or neoadjuvant chemotherapy, or when to consider participation in a clinical trial.

Our analysis identified a surprisingly small number of genes with differential expression between early compared to late stage primary PDAC (Table S1). This finding suggests that primary PDAC may be largely homogenous from a global gene expression standpoint. Nonetheless, the differences that we identified appear to be clinically and therefore biologically important. Our findings of molecular differences in resected primary PDAC tumors suggest that there is subtle biological variation in these tumors that influences outcome. A review of previous published studies did not identify differential expression of our six genes [15],[21],[45]–[56]. This finding is not surprising, as previous studies examined differential gene expression changes between either normal pancreas or chronic pancreatitis and PDAC [15],[45]–[56]. Only one study has looked at gene expression changes between PDAC of different stages [21]. Ours was the first, to our knowledge, to study molecular differences between nonmetastatic versus metastatic primary tumors and identify and validate a prognostic signature for PDAC.

Of the six genes identified in this study, most do not have an obvious role in carcinogenesis. Three of the six genes demonstrated significantly higher expression in the poor prognostic groups (*SIGLEC11*, *KLF6*, *NFKBIZ*; Table S2). *ATP4A*, *GSG1*, and *SIGLEC-11* have not been studied in cancer. SIGLEC-11 is thought to be expressed by tissue macrophages and also the brain microglia [57]. Interestingly, a missense mutation of *SIGLEC-11* (S465A) was identified in the mutation discovery screen of the recent genome-wide sequencing of PDAC [58]. NFKBIZ, also called IkappaB zeta, binds to the p50 subunit of nuclear factor (NF)-kappaB and is important for interleukin-6 (IL-6) induction and may be induced by IL-1 receptor and Toll-like receptors [57]. Given the prevalence of chronic pancreatitis and high degree of stromal fibrosis, it is possible that NFKBIZ may play a role in PDAC and inflammation.

KLF6 is a transcription factor and its full length transcript is thought to be a tumor suppressor gene involved in prostate, lung, and ovarian carcinogenesis [59]. However a splice variant KLF6-SV1 has been shown to have oncogenic properties. The oligonucleotide probes used in the Agilent whole human genome array and the antibody against KLF6 did not differentiate between the full-length and splice variant. In agreement with a previous study [38], we found that KLF6 protein expression was higher in tumors than normal pancreas. In addition we found that higher KLF6 expression was associated with worse survival. Hartel et al. further investigated KLF6-SV1 expression in their study using real-time PCR and demonstrated that the higher KLF6 expression seen in tissues was associated with a higher ratio of KLF6-SV1 compared to full-length KLF6. Therefore our findings that KLF6 expression is higher in tumors and is prognostic is likely in agreement with this study.

Only one patient in the UNC1 cohort was treated with neoadjuvant chemotherapy compared to 80% of NEB patients who were treated with palliative chemotherapy. Although there is a possibility that our signature may be reflective of gemcitabine treatment or perhaps resistance, as NEB patients died of metastatic disease despite gemcitabine treatment, the successful application of our six-gene signature on an independent test set of patients where only 3% of patients with localized PDAC were treated with neoadjuvant therapy suggests that it is a rigorous predictor of prognosis in previously untreated patients. We found no association between our six-gene signature and whether a patient received adjuvant chemotherapy. In addition, chemotherapy treatment in this cohort, either pre- or postoperative, did not demonstrate a survival advantage.

Another concern is the validity of our hypothesis that gene expression changes at different stages of primary PDAC development may occur and be important for prognosis. Our study is in agreement with Lowe and colleagues' findings that differential gene expression changes can be identified within primary PDAC [21]. However, they did not address the prognostic value of their findings. Several studies have also suggested that gene expression changes in metastasis may be found in primary tumors. In a study of molecular differences between primary tumors and metastases, Golub and colleagues identified a gene expression signature of metastasis present that could be identified in primary tumors [60]. In addition, studies in melanoma have suggested that metastatic cells may be found in the parent primary tumor [61]. Finally studies in breast cancer have demonstrated that gene expression changes found in breast cancer cells with metastatic potential may be prognostic and predictive of patients who will develop metastasis [62]–[64]. Our study is the first to demonstrate that molecular differences in metastatic PDAC can be identified at earlier stages, and that these differences are predictive of future behavior. Whether these molecular changes are biologically associated with metastatic potential will require further investigation.

We have applied our six-gene signature to an independent dataset of 67 patients, and have validated its prognostic value. In addition, we have validated the protein expression of KLF6 in a 50-patient TMA. Although not nearly as powerful a predictor of prognosis as our six-gene signature, we found that KLF6 expression was prognostic in our 50-patient TMA. Further validation studies will be needed to see if KLF6 alone may be a useful prognostic marker as others have shown [38]. Our findings suggest that the prognostic value of KLF6 is strengthened in evaluating the six genes in their entirety.

Studies of patients with resectable PDAC demonstrate median survivals of up to 22 mo, equivalent to the median survival of patients in our training and testing cohorts [3],[11],[65]. Our finding that our six-gene signature is able to stratify patients, with startling differences in survival, suggests that it may be used to select patients for therapies. For example, for patients who are at high operative risk, knowledge of a median survival of 49 compared to 15 mo, may be helpful in the operative decision-making process. Similarly, patients who have a poor prognosis based on the six-gene signature may be considered for neoadjuvant therapy. Currently, the minority of centers use neoadjuvant therapy as a standard of care, most instead reserve this for patients with locally advanced unresectable or borderline resectable tumors. Therefore the current decision-making process is based on anatomical considerations. Our prognostic signature may refine this paradigm such that neoadjuvant therapy is offered to patients on the basis of biological considerations, regardless of resectability, and may allow us to further study and maximize the benefits of neoadjuvant treatment. In addition, as new therapies are developed, it may help to determine whether patients may require more or less aggressive treatment. Finally, our findings that there are molecular differences associated with late-stage primary tumors, which translate into differences in prognosis, suggest that the six genes in this signature should be further studied for their potential as biomarkers, and some of these genes, or the pathways that they fall into, may represent new therapeutic targets.

## Supporting Information

Table S1 SAM of metastatic compared to localized primary tumors.
(1.75 MB XLS)

Table S2 Comparison of individual genes in the six-gene signature between the high- and low-risk groups.
(0.03 MB XLS)

## Abbreviations

DWD: distance weighted discrimination
NEB: University of Nebraska Medical Center Rapid Autopsy Pancreatic Program
PDAC: pancreatic ductal adenocarcinoma
SAM: significance analysis of microarrays
TMA: tissue microarray
TNM: tumor, node, and metastasis
UNC: University of North Carolina at Chapel Hill
